# Single center experiences with telemetric intracranial pressure measurements in patients with CSF circulation disturbances

**DOI:** 10.1007/s00701-020-04421-7

**Published:** 2020-06-03

**Authors:** Valentina Pennacchietti, Vincent Prinz, Andreas Schaumann, Tobias Finger, Matthias Schulz, Ulrich W. Thomale

**Affiliations:** 1 Pediatric Neurosurgery, Campus Virchow Klinikum, Charité, Universitätsmedizin Berlin, Augustenburger Platz 1, 13353 Berlin, Germany; 2 Department of Neurosurgery, Charité Campus Mitte, Universitätsmedizin Berlin, Berlin, Germany

**Keywords:** Hydrocephalus, Intracranial pressure, Telemetric, Sensor Reservoir, Neurovent-P-tel

## Abstract

**Background:**

Hydrocephalus may present with heterogeneous signs and symptoms. The indication for its treatment and the optimal drainage in complex cases may be challenging. Telemetric intracranial pressure measurements (TICPM) may open new perspectives for those circumstances. We report our experiences using the Neurovent-P-tel and the Sensor Reservoir in a retrospective study.

**Methods:**

A series of 21 patients (age range 10–39.5 years) treated in our Pediatric Neurosurgical Unit receiving a TICPM was analyzed. In 8 patients, a Neurovent-P-Tel was implanted; 13 patients received a Sensor Reservoir, 6 of which as a stand-alone implant, while 7 were already shunted. TICPM were performed on an outpatient basis. Possible complications, follow-up surgeries, and TICPM were analyzed.

**Results:**

Concerning the complications, one infection was seen in each group and one postoperative seizure was observed in the P-tel group. TICPM-assisted shunt adjustments lead to clinical improvements in six patients in the P-tel group and six patients in the Sensor Reservoir group. In four out of six non-shunted patients, TICPM contributed to the indication toward shunt implantation.

**Conclusions:**

TICPM seems to be a promising tool to improve clinical management of shunted patients with complex hydrocephalus. The two available systems will need further technical improvements, concerning implantation time, measurements, and data analysis in order to optimize handling and interpretation of the data.

## Introduction

Hydrocephalus may present with a great variety of signs and symptoms, generally categorized as acute, subacute, and chronic as well as age-dependent symptoms. Its treatment strategies rely on the possibility to solve the underlying cause for cerebrospinal fluid (CSF) circulation disturbances. However, the necessity of permanent CSF diverting shunts remains the most often treatment option [7, 8, 16]. Different forces influence shunt drainage such as intraventricular pressure, hydrostatic pressure, acting against intra-abdominal pressure, resistance of the valve, and resistance of tubing [11], combined with factors like type of hydrocephalus, ventricular volume, activity of the patient, and body weight [13]. Due to this heterogeneity of factors, the optimal valve adjustment may not be easy to predict [13, 14]. Especially chronic clinical conditions of CSF circulation disturbances such as arrested hydrocephalus, idiopathic intracranial hypertension, or normal pressure hydrocephalus represent some diagnostic difficulties and may require additional invasive diagnostic tools. Hereby, intracranial pressure (ICP) may be monitored via ventricular or lumbar drainage together with possible tests of intermittent CSF release. An external ventricular drain (EVD) or a lumbar drain allows monitoring of the CSF pressure, while also CSF drainage can be established in parallel [23, 24]. The combined evaluation with the clinical status may help for diagnostic decision making. Similar unclear conditions can be found in patients with CSF diverting shunt systems, which do not allow to monitor the drained volume of CSF or the dynamics of ICP. Adjustability of the valve resistance is, however, a tool to influence CSF drainage [4, 12, 18]. This maneuver does not necessarily rely on objective measures and protocols but rather on subjective experience, but may be guided by temporal changes of clinical symptoms, ventricular width in imaging, or head growth in children, as well as on expectancies of patients and relatives. In our previous studies, we have demonstrated that using gravitational-assisted shunt systems, valve adjustments were integrated in the treatment protocol based on guidelines implemented at our institution [13]. Especially in complex clinical conditions, ICP monitoring may help to verify possible clinical improvements achieved by shunt adjustments [4, 12, 18].

The recent introduction of telemetric systems enables to measure ICP (Neurovent-P-tel sensor, Raumedic, Helmbrechts, Germany and Sensor Reservoir, Miethke, Potsdam, Germany) on an outpatient basis in order to objectify uncertain diagnostic conditions in CSF pathologies. In a previously published case series, we demonstrated feasibility of parenchymal Neurovent-P-tel sensor ICP measurements in different positions and that it helped to adjust the valve systems and ameliorate the clinical condition in complex shunted patients [12]. Previously, Juhler and colleagues were investigating posture-dependent ICP changes, age-dependent ICP correlations during daytime measurements, and evaluated therapeutic consequences after telemetric ICP measurements in shunted patients using the P-tel probe [3, 18, 20]. Furthermore, the Sensor Reservoir is integrated in a shunt system to normalize the ICP profile of the patient for clinical improvement of possible under or overdrainage symptoms [4]. With the current manuscript, we report our single-center retrospective experience using the Neurovent P-tel as well as the Sensor Reservoir in patients with CSF dynamic disturbances in a pediatric neurosurgical unit.

## Methods

### Telemetric ICP devices

**Fig. 1:**
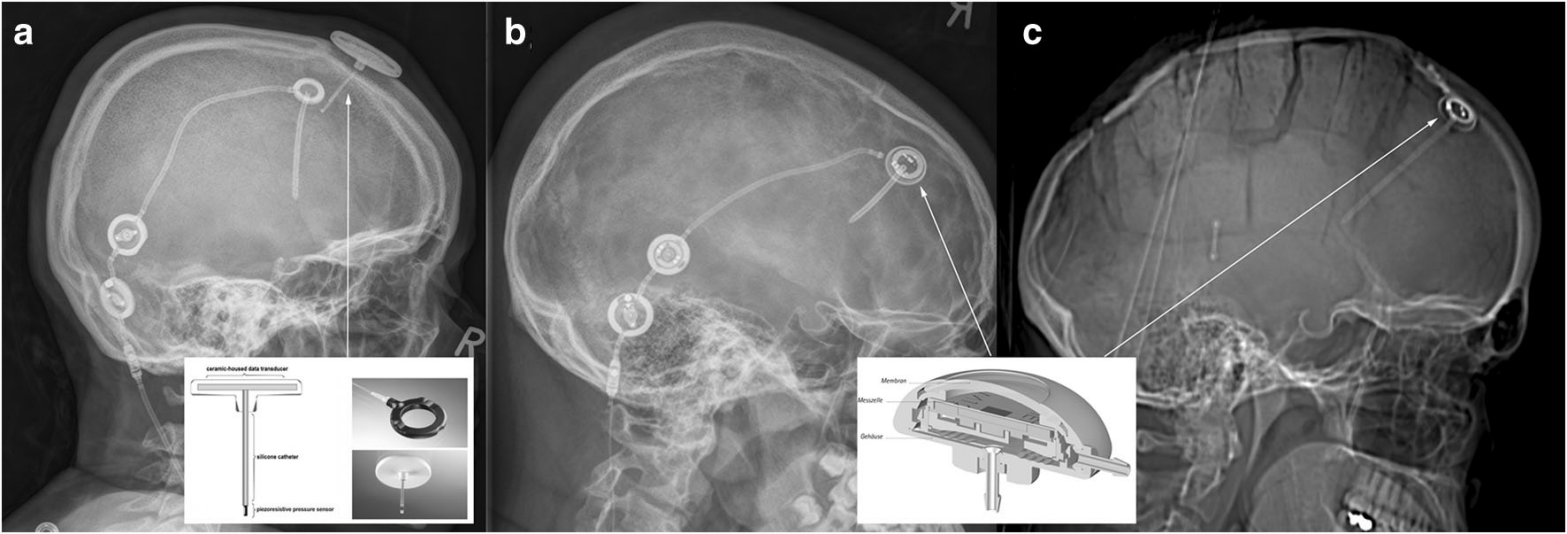
Representative x-rays of the different set ups: **a** AP view on a Neurovent P-tel device on the left (*) and shunt system on the right with a proV-proSA-GA valve complex. **b** Lateral view on a Sensor Reservoir connected with proV-proSA-GA valve complex. In both the proSA was placed in between the two components of a proGAV valve. **c** Lateral view on a Sensor Reservoir as stand-alone device. *proV* programmable DP valve, *proSA* programmable shunt assistant, *GA* gravitational assistant unit, *proGAV* programmable DP valve with gravitational assistant unit

The *Neurovent*-*P*-*tel sensor* (Raumedic, Helmbrechts, Germany) is composed of a silicone-coated catheter (25 mm of length), whose tip contains a piezoelectric pressure sensor and its base, which hosts a disc-shaped data transducer. The base has to be positioned on the skull in the subcutaneous space (Fig. 1a). A radiofrequency transmission coil connected to a portable data recording device allows the acquisition of the ICP values. The pressure sensor is regularly inserted in the frontal lobe parenchyma. If placed in parallel to a shunt, it is positioned on the contralateral side of the ventricular catheter, thus measuring intraparenchymal pressure values. Implantation time should not exceed 90 days according to the company’s recommendations. The P-tel offers to measure pressure values with a detection rate from 1 up to 5 Hz.

The *Sensor Reservoir* (SR, Miethke, Potsdam, Germany) is composed of a titanium-covered measuring cell hosted in a polyether-ether-ketone ring covered with a silicone membrane to allow puncture in order to withdraw CSF. The measuring unit contains a pressure sensor that directly transmits CSF pressure to the titanium cover and thus to the measuring unit. The device can be included in a shunt system and positioned on the burr hole, connected to a ventricular catheter proximally and a subcutaneous catheter laterally (Fig. 1b). To allow correct and reliable use of the device, the catheter position must be purely intraventricular to enable free flow of the water column toward the pressure cell, which was achieved to place the catheter correctly with a ventricular catheter guide [27]. The pressure values represent pressure measurements in the shunt system. The sensor is integrated in the CSF shunt system, which connects the intraventricular cavity with the peritoneal cavity regulated by the valve system distally located to the sensor. Measurements are allowed via a radiofrequency transmission antenna connected to a monitor. The manufacturer does not define an explantation time of the device. The device offers single measurements as well as high- and low-frequency measurements. The Sensor Reservoir offers to measure pressure values with a detection rate up to 44 Hz. This resolution of data acquisition was enabling to detect ICP waveforms during TICPM; however, detailed peak analysis of ICP pulsation could not be visualized.

### Indication for telemetric ICP measurements

The indication for telemetric ICP measurements in shunted patients was complex hydrocephalus cases with chronic symptoms possibly related to non-physiological CSF drainage, in which mechanical shunt dysfunction could be excluded. In those patients regular protocol of valve adjustments as well of several trials of adapting the valve resistance did not lead to clinical enhancement. The Neurovent-P-tel device was implanted in eight patients with the goal to perform ICP monitoring assisted valve adjustments of the preexisting shunt in complex hydrocephalus cases. Similarly, the Sensor Reservoir was implanted in seven patients with preexisting shunts to optimize the valve settings by adding objective pressure assessments to clinical complaints for decision making.

**Fig. 2:**
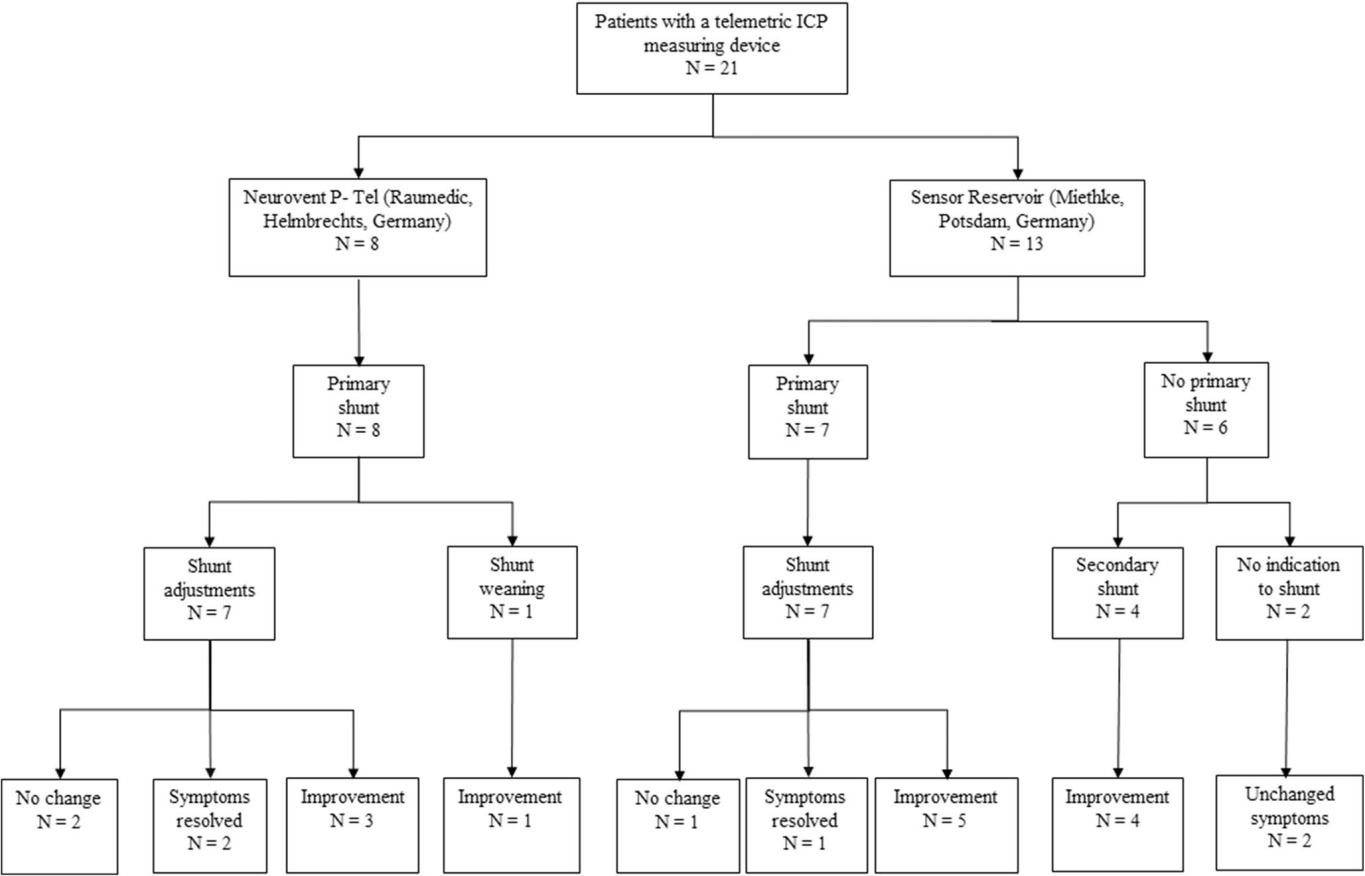
Flow chart showing representing the different groups of treatment strategies with either a Neurovent P-tel or a Sensor Reservoir. The clinical course of the patients with a primary shunt showed clinical improvement in 12 out of 15 patients. When using the Sensor reservoir as stand-alone device for diagnostic purposes in three out of six patients, the indication for a shunt implantation was made resulting in subjective clinical improvement, respectively

The indication for telemetric ICP measurements for diagnostic purposes was unclear chronic clinical condition of suspected increased ICP elevation and possible candidates for CSF shunt treatment. In this context, in six patients, the Sensor Reservoir was used as “stand alone” solution in which a ventricular catheter was placed in the frontal horn of the lateral ventricle [27] connected to the Sensor Reservoir which was filled with CSF and was laterally occluded by a blind connector (Fig. 1c). This enabled not only the measurement of intraventricular pressure but also to diagnostically puncture the reservoir in order to detect pressure changes and possible clinical alterations after CSF volume relieve. This option was chosen in patients with unclear indication for a CSF diverting shunt in order to strengthen the decision for or against shunt implantation. Thereby, repeated lumbar punctures with unreliable single pressure detections were avoided (Fig. 2). Families or caretakers were informed about the risks and benefits of the different treatment options.

### Demographic and clinical data

A total of 21 patients in whom a telemetric device for ICP measurement was implanted in our Pediatric Neurosurgical unit between December 2010 and July 2018. Median age of the population was 16.5 years (range 10–39.5 years); 15 patients were females. In eight patients, a Neurovent-P-tel (Raumedic, Helmbrechts, Germany) device was implanted, while 13 patients received a Sensor Reservoir (Miethke, Potsdam, Germany; see Flow Chart Fig. 2). The Neurovent-P-tel population was treated from December 2010 to March 2015 with a median age of 16.3 years (range 10–21.6 years). Five patients were females. The primary diagnosis was craniopharyngioma in two, Crouzon syndrome in two, and intracerebral gliomas and spina bifida in one patient, respectively. In the Sensor Reservoir group, patients were treated from December 2015 to July 2018, median age was 16.4 years (range 13.1–39.5 years), and ten patients were females. The group included four spina bifida, three posthemorrhagic hydrocephalus, one craniopharyngioma, one craniosynostosis, one traumatic brain injury, one suprasellar arachnoid cyst, one pseudotumor cerebri, and one aqueductal stenosis after ETV. All clinical data is summarized in Table 1.

**Table 1:** Patient demographics and treatment regimens

	P-Tel (Raumedic)	Sensor Reservoir (Miethke)	
Number of patients (*n*)	8	13	
Median age (range) (years)	16.3 (10–21.6)	16.4 (13.1–39.5)	
Male:female (*n*)	3:5	3:10	
Diagnosis (n type)	3 gliomas	2 spina bifida	2 spina bifida
	2 craniopharyngiomas	2 PHH	1 craniosynostosis
	2 Crouzon syndromes	1 craniopharyngiomas	1 SS arachnoid cyst
	1 spina bifida	1 TBI	1 IIH
		1 acqueductal stenosis after ETV	1 PHH
No primary shunt (*n*)			6
Primary shunt (*n*)	8	7	
Type of shunt (*n* type)	6 proGAV/proSA	4 proGAV/proSA	
	1 proGAV/proSA/SA	2 proGAV 2.0	
	1 proV/proSA	1 proV/proSA	
Explantation (*n*)	6	1	
Complications (*n* type)	1 infection	1 infection	
	1 seizure/ edema		
Mean amount of further surgeries (range) (*n*)	1.5/6 (0–4)		
Surgical outcome (*n* type)	1 shunt explantation		4 shunt implantations
Clinical outcome (*n* category)	2 resolved	1 resolved	
	4 improved	5 improved	4 improvements
	2 unchanged	1 unchanged	2 unchanged

*proGAV* adjustable differential pressure valve with gravitational assitant, *proSA* adjustable shunt (gravitational) assistant, *SA* shunt (gravitational) assitant *proV*, adjustable differential pressure valve, *PHH* posthemorrhagic hydrocephalus, *TBI* traumatic brain injury, *ETV* endoscopic third ventriculostomy; *SS* suprasellar, *IIH* idiopathic intracranial hypertension

### Study-protocol

Telemetric ICP measurements were performed on an outpatient basis. A standard sequence of body postures, namely standing, lying, and sitting, was carried out during measurements. The lying position was adjusted with a cushion of 15 cm height under the head to avoid a Trendelenburg position. Measurements were repeated four to six times per positioning as single measurements in order to calculate the mean ICP. For optimizing shunt adjustments, the aim was to balance the ICP values according to the following target ranges. In standing and sitting position, an ICP was aimed to be between − 10 and 0 cmH_2_O, while in the lying position the targeted range was at 5 and 15 cmH_2_O. In case of lower ICP in lying position, the resistance of the differential pressure (DP) valve was adjusted to a higher resistance; in case of lower ICPs in upright position, the gravitational unit was adjusted to higher levels or vice versa, respectively. Normally, each setting change included two points on the DP unit or up to four points on the gravitational unit. The patients were asked to write a diary including headache intensity (visual rating scale 0–10) and activities. During the course of adjustments, the subjective patient’s complaint alterations were categorized based on the following three states: unchanged symptoms, improvement, or resolved symptoms. The pressure values were given in cmH_2_O, consistent with the resistance settings as used in the gravitational-assisted valves (Miethke, Potsdam, Germany).

In six patients, the Sensor Reservoir was implanted as “stand-alone” solution for diagnostic purposes (Figs. 1c and 2). An informed consent was collected for an individual treatment option and the risks and benefits of this invasive procedure as compared to repeated lumbar punctures were thoroughly discussed with patients and caregivers. In these patients, TICPM were performed using the same protocol of body posture changes in the outpatient clinic. In addition, after at least two appointments for TICPM, the reservoir was punctured and a CSF volume of about 10–20 ml was withdrawn. The sampling process was interrupted if the patient complained about any worsening of symptoms. After CSF relieve, another measurement was performed in the same manner. The indication for shunt implantation in those patients was established according to the following criteria: ICP values in lying position > 20 cmH_2_O and in standing position > 0 cmH_2_O and symptom amelioration after CSF subtraction by reservoir puncture. If a patient showed repeated clinical symptom amelioration after CSF relieve together with decrease in telemetric ICP values after reservoir puncture independent from ICP guidelines, a shunt therapy was also discussed with the patients or families.

In all cases, an informed consent, signed by the patients and/or their parents or legal representative, was collected before proceeding to any surgical or other intervention. In the case of the Neurovent-P-tel, when the manufacturer’s certified implantation time expired, a recommendation for the explantation of the device was given to the patient or family. However, the decision to consent for this additional procedure was left to patients or families. For safety measures, we documented any implantation-related complications of the telemetric ICP devices. Further surgeries to the implanted shunt systems were recorded during further follow-up.

### Statistical analysis

Values are given as median with range or mean and standard deviation. For comparison of multiple ICP measurements in different body postures and different patient conditions, a one-way ANOVA was used followed by Tukeys multiple comparison test to detect possible differences among the variables. Possible correlation among two parameters were evaluated using a linear regression analysis. For implant revision-free survival analysis, a Kaplan-Meier curve was established. Statistical significance was considered at *p* < 0.05. Statistical analysis was performed using Prism 7 software (GraphPad, San Diego, CA, USA).

## Results

### Clinical course of patients

### Perioperative course of the Neurovent-P-tel group

The valve combinations chosen among the eight patients with a Neurovent-P-tel was in six cases a proGAV/proSA, in one a proV/proSA valve, and in one a proGAV/proSA/SA (Table 1). After implantation of the ICP Sensor, a total of six additional surgeries to the shunt became necessary in three patients, which included one subcutaneous kinking of the catheter after proSA augmentation; one valve augmentation with a shunt assistant together with a laparascopic inspection and bactiseal catheter replacement in another case; a valve augmentation with a shunt assistant, an intraoperative shunt test, externalization due to potential dysfunction, and finally reconnection of the shunt system in the third case. One shunt could be completely weaned and was explanted after changing the valve to maximal settings of 20/80 cmH_2_O and ICP values in the predefined range which was considered to be normal. The remaining headaches were not related to the shunt but were still better than before shunt explantation and were further treated conservatively (Fig. 2). During further follow up after TICPM, additional shunt revisions were necessary including three valve exchanges and one subcutaneous tube kinking which lead to shunt malfunction.

As complications of the telemetric ICP sensor, one case developed an infection of the device at 1.6 months, and in another patient a seizure was observed which correlated with minor bleeding and edema at the frontal parenchymal site insertion at 12 days after implantation. Both were explanted earlier as planned. In further clinical course, the explantation of the system was planned in four cases according to the specification of the manufacturer, while two patients refused an additional surgical intervention and anesthesia for explantation of the probe. The median implantation time of all implants was 4.2 months (range 4–13 months).

### Perioperative course of the Sensor Reservoir group

For the shunted patients in Sensor Reservoir group, the valve combination included four proGAV/proSA, two proGAV 2.0, and one patient with a proV/proSA. For the cases with stand-alone Sensor Reservoir implants, four out of six patients received a VP shunt of whom two received a proGAV 2.0 and one received a miniNAV/ proSA valve combination. During follow-up, one of the patients needed a valve exchange with higher gravitational resistance.

**Fig. 3:**
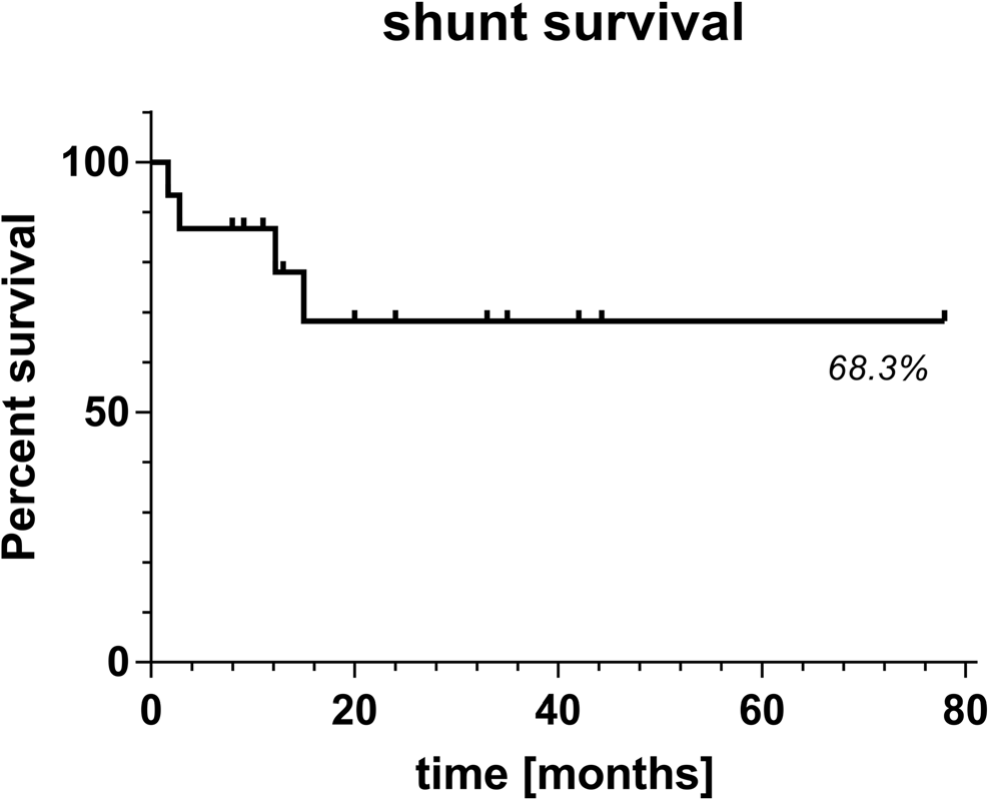
KaplanMeier analysis for shunt revision free survival after implantation of telemetric ICP monitoring device

In the Sensor Reservoir group, one patient required unintentional explantation due to infection 23 days after implantation. This case had been revised repeatedly earlier due to infection of cranioplasty implants as well as shunt revisions. All other Sensor Reservoirs are still in place during a total follow-up time of 35.3 months.

#### Clinical Follow-up

**Fig. 4:**
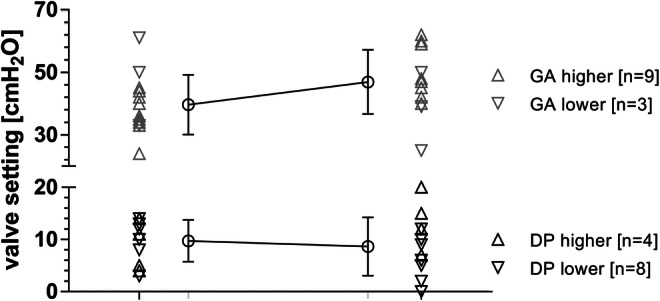
Telemetric ICP assisted valve setting changes. The majority of differential pressure (DP) valve setting changes mainly for horizontal position was lowered while the majority of gravitational (GA) valve settings for the vertical position was increased

The shunt revision-free survival after TOCPM for the entire cohort and both devices was 68.3% following 77.9 months of follow-up (Fig. 3). Looking at the outcome of the entire TICPM cohort, the symptoms remained mainly unchanged for three patients in which the explantation was performed unintendedly. For all patients who received long-term ICP-assisted valve adjustments, the clinical complaints were enhanced after balancing the ICP values in the body postures by shunt adjustments, respectively. This resulted in no headaches for three patients and improvement of symptoms for nine patients. During this treatment regimen, the valve settings were adjusted toward higher resistance for the lying position in four patients and toward lower resistance in eight patients, while the adjustments reached higher settings in vertical position in nine patients but reached lower settings in two patients, while no change in vertical position was performed in one patient (Fig. 4).

In four out of six patients with a stand-alone Sensor Reservoir, a shunt implantation led to amelioration of symptoms in all patients and resolving symptoms in two patients. In the two remaining patients, who did not receive a shunt, no changes in clinical symptoms were observed after CSF puncture as well as no clear pathological ICP values could be detected which indicated shunt surgery (Fig. 2).

### ICP measurements in different body postures

Given ICP values were taken from Sensor Reservoir patients. As reported earlier, the TICPMs showed significant lower values in standing and sitting position compared to lying position (*p* < 0.0001). In standing position, the mean ICP is − 3.6 ± 5.1 cmH_2_O (range − 14.2–6.8 cmH_2_O), in sitting − 3.7 ± 4.4 cmH_2_O (range −12–4 cmH_2_O), and in lying position ICP 14.83 ± 7 cmH_2_O (range 2.6–26.98 cmH_2_O).

In patients with shunt compared to no shunt receiving the Sensor Reservoir for diagnostic purposes, the ICP values differed significantly among the groups in standing (*p* < 0.0001) and in sitting position (*p* < 0.001) but not in lying position. Patients with no shunt had a mean ICP in standing position − 3.6 ± 5.1 cmH_2_O, in sitting position − 3.7 ± 4.4 cmH_2_O, and in lying position 14.8 ± 7cmH_2_O, while shunted patients ICP was in standing position − 12.2 ± 5.5 cmH_2_O, in sitting position − 10.8 ± 5.8 cmH_2_O and in lying position 11.5 ± 6.9 cmH2O.

In four patients with ICP measurements for diagnostic purposes who finally received a VP shunt, pre- and post-shunt ICP measurements could be evaluated. In those reduced ICP, values could be detected in upright and lying position after shunting reaching statistical significant difference only in the lying position (*p* < 0.05). ICP values before shunt in standing position were − 2.2 ± 6.5 cmH_2_O, in sitting position − 4.4 ± 5.4 cmH_2_O, and lying position 18.2 ± 8.3 cmH_2_O, while ICP values after shunt was measured in standing position − 11.6 ± 4.7 cmH_2_O, in sitting position − 10.1 ± 4.6 cmH_2_O, and in lying position 7.5 ± 3.8 cmH_2_O.

### Case example with correlation of ICP changes with valve adjustments

In a 13-year-old boy with posthemorrhagic hydrocephalus after prematurity, chronic headaches were reported. The patient had primarily a paediGAV (9/29 cmH2O) since early childhood and had no shunt revision until the age of 12 years. At this age, he reported about headaches related to activity, more pronounced later during the day rather than in the morning. Thus, the symptoms were interpreted as overdrainage-related headaches. The patient received a proGAV with augmentation of a proSA in order to achieve higher resistance especially in upright position. However, this did not initially lead to clear changes in clinical complaints. Even after implantation of a Sensor Reservoir, higher valve resistance and better balance of ICP values improved the symptoms but did not lead to resolution of headaches. Hence, the patient demanded the stepwise adjustment of the valve toward the paediGAV setting in which he subjectively believed he would have less symptoms. This resulted, however, to dramatically negative ICP values even measured in the lying position. The headaches worsened and the patient finally agreed on readjusting the valve to higher settings again reaching a clear amelioration of the headaches. Remaining symptoms were subsequently treated conservatively with pain medication, psychological consultation, and stress relieve from school demands.

**Fig. 5:**
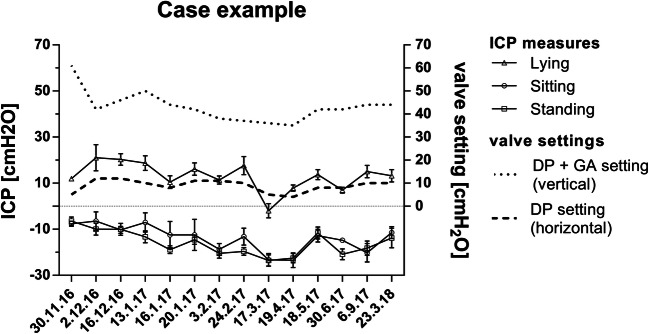
Representative case showing the association of telemetric ICP measurements in lying, sitting, and standing position, together with the respective valve settings over time. The valve setting in horizontal position is determined by the differential pressure (DP) valve alone, while the valve setting in the vertical body position is determined by adding the differential pressure (DP) and the gravitational pressure (GA)

The time course of adjustments and TICPMs are depicted in Fig. 5. These intensive TICPMs and multiple valve adjustments allowed to analyze the relationship between valve settings and TICPMs. First of all, it was possible to prove that valve setting adjustments lead to changes in three-positional TICPMs correlating to clinical complaints as described above. Correlating ICP values of each body posture to the valve adjustments it was shown that ICP values in all body positions significantly related to differential pressure settings (proV) of the valve (*p* < 0.05). The highest correlation coefficient was shown in the lying position (*p* < 0.0001). In contrast, ICP values in the standing and sitting position correlated significantly with the adjustment changes of the gravitational valve (proSA) (*p* < 0.01), but not in the lying position (*p* = 0.8).

## Discussion

The present study reports about a cohort of patients treated in a pediatric neurosurgery department, including children and young adults from the transitional patient cohort with difficult-to-treat hydrocephalus cases. In those patients, a combination of unsolved clinical symptoms possibly related to non-physiological CSF drainage, mostly chronic or recurrent headaches, after exclusion of shunt malfunction by sometimes multiple surgeries for valve exchange or augmentation and repeated adjustment of the valve resistance without clear clinical benefit. Thereby, it was feasible to objectify whether or not a relationship existed between the clinical complaints and the hydrocephalus conditions.

### TICPM in shunted patients

So far, only 1 case series describes the experience with 25 shunted patients who underwent a Sensor Reservoir implantation [4] to monitor ICP in order to reach better valve and gravitational unit settings. An ICP-guided valve adjustment brought a clinical improvement in the majority of cases (70%), with an evident change of the ICP values in both horizontal and vertical positions; however, no revision-free survival rates are reported. The age range of this population was 13–81 years (mean age 53.6 ± 20.7 years). Another recent study showed an age-dependent linear decrease of the daytime ICP values (overall decrease from 4 to 85 years of age was 5.6 mmHg). The difference between day and night ICP in children was significant, with a higher ICP at night referring to more pronounced lying positions. On the contrary, nighttime and daytime ICP were found to be higher in children but did not reach significant difference in adults [20]. Lilja et al. (2014) reported their experiences in the management of 22 complicated cases of impaired CSF circulation, using the Raumedic telemetric ICP probe (Neurovent-P-tel). Implantation time was limited in only four patients and the reading period was < 6 months in 12 patients. The authors concluded that one-third of the recording sessions was directly followed by a surgical shunt revision [18].

Until today, only limited data concerning hydrocephalus originating in pediatric age have been published yet. Barber et al. [5] reported their clinical and economic considerations on their experience with a purely pediatric cohort of four patients (4–16 years), implanted with a Neurovent-P-tel device (time of implantation was 460–632 days). Antes et al. also reported a large 5-year experience with Neurovent-P-tel, describing 247 patients, including 10 patients younger than 18 years of age (implantation period 3–90 days) [4]. A large population has been more recently characterized by Norager et al. including 119 Neurovent-P-tel devices in a series of patients from 2 to 80 years of age (mean age: 30 years) [19]. A possible limitation of the presented study is the rather small cohort of patients included, which restricts the ability to draw valid conclusions from the collected data. This circumstance depends mainly on the fact that TICPM is currently time consuming and still in a developmental stage and it was only used in cases which were considered to be complex hydrocephalus cases in terms of optimal valve adjustments or difficult indications for possible shunt treatment. It is also reflected by the fact that from a total of approximately 120 shunt surgeries per year in our institution, only 21 patients received a TICPM in 8 years. In terms of complications with the telemetric ICP devices, we have seen two infections and one edema-associated seizure. Although infection will not necessarily be associated with the specific device but rather with the surgery itself, the complication rate is not low in the presented series. It must be concluded that TICPM adds further surgical risk to the patients; however, if the technique will be used more often in a routine cohort of patient, complication rate may be lowered, prospectively. This will also hold true for shunt revision rate after TICPM. Parts of the described revisions were planned and were direct consequences of ICP measurements. The differences of shunt revision rate beteween the groups are, thus, more likely due to a learning curve, better predicting over time the possible valve combinations in advance rather than linked to the TICPM devices themselves. Finally, the revision rates are comparable to other previously published cohorts using similar shunt protocols [1, 26].

### TICPM devices

Telemetric methods for ICP monitoring have been introduced in the clinical practice nearly 20 years ago [22], such as the “Osaka Telesensor” [15] and the “Codman Microsensor” [6, 9]. So far, there are only few reports about TICPM devices in shunted patients. The currently available TICPM systems are the Neurovent-P-tel and the Miethke Sensor Reservoir, which present some differences regarding the technical set-up and surgical options. While in the Neurovent-P-tel the pressure sensor is located at its tip and thus measures ICP in the brain tissue, the Sensor Reservoir measures the ICP in the reservoir, which is connected to a ventricular catheter and is located on the calvarium, which might lead to relatively lower pressure values according to hydrostatic forces within the ventricular catheter. On the other hand, in order to optimize the valve settings in shunted patients, the Neurovent p-Tel has to be implanted on the contralateral side with an additional approach. Furthermore, the implantation time of Neurovent-P-tel is limited to 90 days by certification. This might add another surgical intervention for explantation, while the Sensor Reservoir can remain in place. So far, most of the series using the Neurovent-P-tel apply longer implantation times, e.g., 208 days in a recent large study [19], since the patients or parents still need to consent for an additional surgery for explantation [17, 18, 29]. The Sensor Reservoir opens the possibility of direct CSF relieve by puncture which was experienced to be advantageous especially in a stand-alone implant. However, the monitoring and ICP analysis software is better evolved for the Neurovent P-tel device, since the Sensor Reservoir offers only the possibility to measure ICP and visualize a pressure curve, which is more detailed with an acquisition rate of 44 Hz compared to 5 Hz in the P-tel device (Table 2).

**Table 2:** Overview of factors comparing both TICPM devices

Specification	Neurovent-P-tel	Sensor Reservoir
Site of implantation	Contralateral to shunt	Integrated in shunt
Sensor location	Intraparenchymal	Integrated in reservoir
ICP measurement	Intracranial	In reservoir/ shunt
Certified period of use	90 days	Unlimited
Detection rate	5 Hz	44 Hz
CSF/tap test	Not possible	Via reservoir
Analysis software	Evolved	Measurement only

### Body position-related ICP measures

Similar to other groups, we experienced that ICP values show a great variability in the first 20° of body posture angulation, basically in the range from lying to sitting posture being negative toward the upright position as reported earlier [21]. Andresen et al. published a paper in which adult patients after small focal brain tumor resection also underwent the positioning of a telemetric ICP monitoring device for research purposes (using Raumedic, Neurovent-P-tel), thus providing an indicative range of values for a normal ICP [3]. Normal values seemed to be lower than in other previous studies and negative values can be accepted as normal especially in upright position, as confirmed by the same group [2]. In a pilot study by Ertl et al., the use of Sensor Reservoirs in line with a shunt system in two cases of NPH also corroborates the concept that negative ICP values in orthostatic positions are a frequent finding [10]. Negative pressure values in upright position are a well reproducible phenomenon also seen in all patients in our series. It is hypothesized that negative pressure may be related to venous outflow in an upright position reducing intracranial blood volume. The pressure changes may be regulated by jugular vein collapse in positions higher than 20° of body position to reduce further venous outflow. Initially, it was experienced as a challenge to correctly interpret ICP values in different body positions and draw the right conclusions for adjusting the shunt valves, accordingly. A reliable understanding developed over time, that the combination of differential pressure together with gravitational pressure valve adjustments opened the possibility to influence ICP values in horizontal and vertical position differently. The differential pressure of valves is active in all body positions, while the gravitational pressure of the valves influences the resistance of the shunt only in the vertical position. Thus, we concluded that the ICP being too negative in vertical position can better be influenced by the gravitational valve setting, while the pressure setting being too positive in the lying position can better be influenced in the differential pressure valve setting and vice versa. That is especially important since the measured ICP in standing position is significantly higher than the valve adjustment (DP + GA pressure) in order to overcome the hydrostatic effect sufficiently. For future development, it will remain a huge challenge that future studies will need to evaluate the normal ranges of ICP related to body position, respectively.

### ICP curve analysis for future development

Even if we have used some guideline parameters for shunt indication in our diagnostic group, we also experienced patients with lower values, who did profit from shunting after repeated symptom amelioration following taping the reservoir. In those patients, the pre ICP measurement seems to be insufficient and compliance evaluation may be of higher importance. At the current state, telemetric ICP monitoring allows mainly mean ICP measurements in both devices and ICP curves visualization in Sensor Reservoir but excludes more detailed analysis of an individual ICP pulse wave. In fact, long ICP monitoring shows quiet a complexity of values with sometimes a large amplitude variability in comparison to single sporadic ICP measurements [23]. It was recommended that ICP monitoring should be performed overnight or for a minimum of half an hour in the supine position [28]. Recently, ICP amplitude was discussed to play an important role for evaluating intracranial compliance. For this reason, ICP pulse wave amplitude (AMP) and the RAP index (describing the cerebrospinal compensatory reserve, derived by the linear correlation coefficient between AMP and the mean ICP between 40 consecutive, time-averaged data points) seem to be more reliable parameters in evaluating shunted patients. It is discussed that a limited compliance is shown by higher ICP amplitude, while a RAP > 0.6 describes a low compensatory reserve or compliance [25]. These parameters have been evaluated after overnight monitoring of the ICP and during infusion study tests [25, 28]. Hence, further future developments of ICP data analysis are needed for telemetric ICP monitoring to integrate more elaborated ICP waveform analysis parameters such as amplitude and waveform characteristics in an automatized analysis software in order to make the devices more intuitive and time efficient in terms of better data interpretation**.**

## Conclusion

The telemetric measurement of ICP not only allows an ICP-controlled shunt adjustment but also may constitute a valuable diagnostic tool to assess the indication for shunt implantation. The introduction of telemetric ICP devices is currently used in some specialized centers on complex cases, but it does not constitute a neurosurgical routine, yet. From our limited experience, we conclude that TICPM enables the surgeon to better understand the pathophysiology in single patients with a shunting system and may help to better treat and diagnose CSF dynamics pathologies. Its application on shunted patients may assist subsequent tailored adjustments. Its risk/benefit ratio must further be defined in bigger studies. Further improvements are still needed to introduce its regular use in more patients, both concerning the size of the device, as well as data management.
